# An innovative tool to assess the functional resilience of a school system: learning from the COVID-19 pandemic

**DOI:** 10.3389/fpsyg.2023.1291621

**Published:** 2023-11-24

**Authors:** Arielle Kaim, Maya Siman-Tov, Bruria Adini, Shahar Lev-ari

**Affiliations:** ^1^Department of Emergency and Disaster Management, School of Public Health, Faculty of Medicine, Tel Aviv University, Tel Aviv, Israel; ^2^Department of Health Promotion, School of Public Health, Faculty of Medicine, Tel Aviv University, Tel Aviv, Israel

**Keywords:** functional resilience, schools, system, COVID-19, public health

## Abstract

**Introduction:**

Preparing the school system for a future crisis requires the ability to examine the effectiveness of schools’ functioning during distant learning and their level of preparedness for future crises. Functional resilience (FR) is defined as the ability to maintain vital operational continuity in the face of disturbance. The study objectives included to develop a FR index of schools and to evaluate and validate it.

**Methods:**

To enable examination of the study objectives, the study design included tool development, followed by a validation process among 20 content experts. Concurrently, an eDelphi process for building an inclusive index, based on various components of resilience was conducted. The final study tool consists of four tailored questionnaires to examine perceptions of key stakeholders, i.e.- teachers, principals, parents, and highschool students regarding communication, psychosocial aspects, perceived stress, infrastructure, resources, pedagogic support, digital literacy, and perceived FR. Using an internet panel, the tool was disseminated cross-sectionally among the four groups of stakeholders.

**Results:**

The results showed high reliability of most of the scales developed. Furthermore, a high consensus level was reached on the relative importance of each component/ stakeholder to the schools FR. The findings further suggest that there were no significant differences in the composite FR score based on characteristics such as school type/ size/geographic location. However, the findings revealed interesting variations among stakeholders, with findings suggesting greater vulnerability among some.

**Discussion:**

To increase resilience and preparedness for future adversities that school systems may face, it is recommended to periodically incorporate an assessment based on a structured tool.

## 1 Introduction

During the COVID-19 pandemic, governments worldwide, including Israel, implemented school closures as a response (Paltiel et al., 2021). This measure resulted in the suspension of face-to-face learning and the transition to virtual education (Daniel, 2020; Donitsa-Schmidt and Ramot, 2020; Stein-Zamir et al., 2020). The fluctuating course of the pandemic has led to repeated closures and the adoption of distance learning as a crisis management measure due to new lockdowns and the emergence of more contagious variants (UNESCO, 2020). These closures have affected a significant majority of the world’s 1.6 billion schoolchildren, with some countries experiencing up to 60 weeks of shutdowns (Contini et al., 2021). According to estimates by the World Bank, a 5-month school closure could result in a staggering $10 trillion in learning losses (Azevedo et al., 2021; Psacharopoulos et al., 2021).

The most vulnerable children, who often depend on schools for their educational, nutritional, and health needs due to socioeconomic disadvantages or disabilities, have borne the brunt of these temporary shutdowns (Colao et al., 2020). Reports from the United States during the COVID-19 crisis have highlighted significant disparities in access to quality educational instruction, digital technology, and internet connectivity. Students in both rural and urban school districts have encountered challenges in accessing the internet, with as many as one-third of students in some urban areas unable to participate in online classes (Dooley et al., 2020). Consequently, a substantial group of schoolchildren continues to be excluded not only from learning but also from socializing with their peers.

The impact of school closures extends beyond students, affecting various stakeholders such as school staff, administration (including principals), teachers, and parents (Grooms and Childs, 2021; Lugo, 2022). The transition to online teaching required teachers and administrators to adapt their instructional methods, particularly for those with limited experience in online pedagogy, necessitating the adjustment to digital platforms (Pollock, 2020). These changes resulted in teachers reporting a significant increase in their workload, feeling socially isolated from colleagues and students, and facing challenges in balancing teaching responsibilities with caring for their own children (Flack et al., 2020; Kaden, 2020). Similarly, school principals experienced a notable increase in their workload (Flack et al., 2021).

In the family context, schools play a crucial role in safeguarding and supervising children, enabling parents to work. However, when schools are closed, parents often face the dilemma of either staying at home, leading to potential economic consequences, or leaving their children unsupervised (Armitage and Nellums, 2020). As a result of the COVID-19 pandemic, education has shifted from the traditional classroom setting to the home, placing an additional educational responsibility on parents, albeit to a partial extent (Doyle, 2020).

The COVID-19 pandemic has sparked increased scholarly interest in the concept of “resilience,” examining resilience at various levels, such as the individual, community, national, organizational, and systemic levels (Bryce et al., 2020; Cusinato et al., 2020; Prime et al., 2020; Tso et al., 2020; Zadok-Gurman et al., 2021). Masten (2018) defines resilience as the capacity of a dynamic system to successfully adapt to disturbances that threaten its function, survival, or development (p. 187). Recent resilience research, historically rooted in theory, is now moving toward more operational and evaluative approaches. While there’s a rich body of literature providing conceptual definitions of resilience, there remains a notable gap of operational and institutions definitions for resilience. Within the broader concept of resilience, there is a growing focus on functional resilience, which emphasizes the practical aspects. Functional resilience refers to a system’s ability to withstand, absorb, and respond to shocks or disturbances while maintaining its critical functions, and ultimately recovering or adapting to new circumstances (Biggs et al., 2012; Zhong et al., 2015). In essence, while resilience looks at the overall adaptability and recovery, functional resilience zeroes in on the pragmatic component of ensuring core functionalities remain uninterrupted and perhaps even enhanced post-disturbance. Although research on functional resilience in the education system is limited, insights can be drawn from the context of the health system. In the hospital setting, factors considered in assessing functional resilience include the vulnerability of structural and non-structural components, critical infrastructure, potential impacts on staff and occupants, involvement of external stakeholders, and policies to mitigate adverse effects (Mahmoudi and Mohamed, 2018a). On a systemic level, it is crucial to engage and evaluate stakeholder involvement to ensure alignment with the system’s objectives and goals (Rautela et al., 2011; Loosemore et al., 2013; Mahmoudi and Mohamed, 2018b). However, existing literature on functional resilience suggests a limited investigation into its application on a systemic or institutional level. Therefore, there is a need to develop a comprehensive set of metrics and relevant indicators to assess functional resilience more effectively.

The global closures of schools during the COVID-19 pandemic have exposed the vulnerability of education systems (Krishnamoorthy and Keating, 2021; Leo et al., 2021; Manivannan et al., 2021). The disruption and subsequent reintegration of the schooling system worldwide highlight the importance of ensuring functional resilience in schools during both in-person and distance learning. Therefore, it is crucial to understand the factors that affect the functional resilience of this system. There is a growing recognition that the current crisis presents an opportunity for transformative changes in the education system, with COVID-19 serving as a catalyst for significant reforms (Zhao, 2020). However, there is currently a lack of benchmarks to assess whether these changes will effectively lead to the desired outcomes for schools in Israel and globally. In light of this, the objective of this study was to develop an innovative index, scientifically grounded, called the Functional Resilience of Schools Index. This index aims to evaluate the preparedness of the school system for future challenges, based on the complexities and difficulties identified during and following the coronavirus pandemic. By using this index, it will be possible to measure functional resilience over time, identify institutions in need of support, and consistently enhance the functioning level of schools.

## 2 Methods

An extensive literature review was conducted to determine the components relevant to the functional resilience of various systems, with emphasis on the educational system. Based on the knowledge acquired, a structured tool to assess the functional resilience of schools was developed, combining all components that were found to be relevant for the four stakeholders of the education system, including students, parents, teachers, and principals. Initially, the composite index included 10 categories, and did not include the perceived functional resilience index score. This category was later added, following studies indicating that perceptions play an important part in systemic resilience (Gröschke et al., 2022). Thus, following additional assessment of Alpha Cronbach both before inclusion (*α* = 0.726) and after inclusion (*α* = 0.925), the perceived functional resilience index was integrated into the composite score.

**Figure 1:**
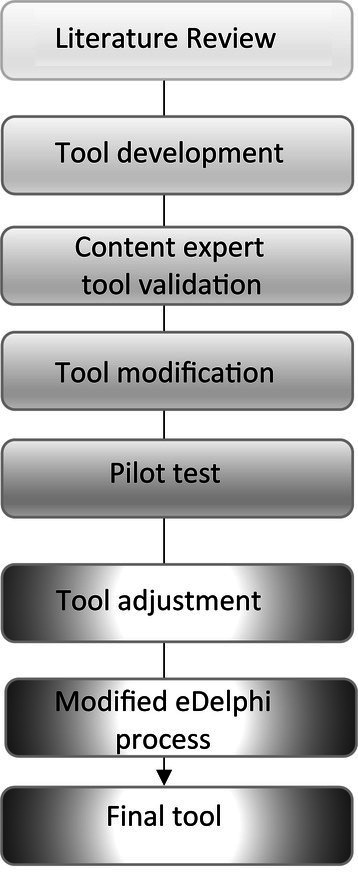
Process of developing and validating the school functional resilience tool.

To ensure the content validity of the tool, the questionnaires were disseminated to 20 leading content experts from the fields of education, resilience, and psychosocial realms, from both the field and academia. The content experts were requested to state their opinions regarding the relevance of each item to the various components determined to be included in the assessment tool of school functional resilience and suggest modifications as they see fit. On the basis of these recommendations, revisions were made to the preliminary tool. Subsequently, the tool was pilot tested among 25 individuals. Following further suggestions made as a result of the pilot (e.g., shortening of the tool, rewording, etc.) adjustments were further made to the tool. Consequently, the revised final tool was disseminated to the respective stakeholders. Simultaneous with the dissemination of the final tool, a modified eDelphi process was conducted with the 20 invited content experts, for the following issues: their perception of the relative importance of each of the components to the functional resilience index (adding up to 100%) as well as the relative importance of each of the stakeholders (adding up to 100%) to the functional resilience index. The level of consensus between the various content experts was compared. The required level of agreement between the experts was predefined as 70% or higher. The process of the study is described in Figure 1.

After the final development of the tool, it was distributed cross-sectionally in October–November 2022, approximately two and a half years following the initial closure of in-person learning in the Israeli school system on March 15, 2022. The research enlisted a comprehensive group of 1802 individuals, encompassing the primary stakeholders in the education system: 1000 students in grades 10 through 12, 301 parents, 449 teachers, and 52 principals, all hailing from 890 high schools within the Israeli Jewish community (Kaim et al., 2023). This sample dataset is the same as in the earlier research conducted and reported in Kaim et al. (2023). Participants voluntarily agreed to take part in the study. The data collection process was conducted by iPanel, the largest internet panel company in Israel, with a membership of more than 140,000 panelists who represent diverse demographic and geographic segments.^1^ iPanel adheres to the stringent guidelines established by the European Society for Opinion and Marketing Research (ESOMAR) for its online platform. The study received ethical approval from the Ethics Committee of Tel Aviv University (number 0004549–1, dated February 13th, 2022) and the Ministry of Education (number 12379, dated April 28th, 2022). Data collection was conducted anonymously.

For statistical analysis, the level of consensus among the content experts was assessed by calculating the percentage of agreement for each category/stakeholder and determining Fleiss kappa for overall agreement. A predefined threshold of 70% or higher agreement was set for each category examined. The weighted functional resilience score was computed using the average score achieved after reaching the predefined level of consensus regarding the relative importance of each stakeholder/component to the functional resilience of schools. Descriptive statistics, including measures like frequency, mean, and standard deviation, were employed to depict the demographic traits of the participants within each of the four stakeholder groups. These same descriptive statistics were also applied to delineate the features of the sampled schools, specifically in terms of percentages, and to assess the distribution and central tendencies of the five indexes. To gauge the differences among the stakeholders, a one-way ANOVA test was applied, and subsequent to that, a *post hoc* Bonferroni test was conducted to pinpoint distinctions among the various groups. Pearson correlation tests were also carried out to examine the relationships between the variable indexes. All statistical analyses were executed using SPSS software version 28, with a predetermined significance level of *p* < 0.05.

## 3 Results

### 3.1 Tool structure

Following the literature review and the validation process, the functional resilience assessment tool was developed, consisting of the following scales:

The evaluation of communication during distance learning involved the assessment of 3 items for students, 5 items for parents, and 7 items for teachers and principals. These items gauged the attitudes regarding the effectiveness of communication during distance learning across all the stakeholder groups. Notably, three of the questions were identical, albeit tailored to suit each specific population. The reliability of the scale was determined using Cronbach’s Alpha, yielding the following results for each of the four stakeholder groups: *α* = 0.833 for students, *α* = 0.887 for parents, *α* = 0.861 for teachers, and *α* = 0.764 for principals.

The evaluation of communication during frontal learning involved the assessment of 1 item for students, 2 items for parents, and 4 items for teachers and principals. These items measured the perceptions of how effectively communication was handled during in-person learning for all stakeholders. Importantly, one of the questions was identical, albeit tailored to each specific group. The scale’s reliability was assessed using Cronbach’s Alpha, yielding reliability coefficients of *α* = 0.726 for parents, *α* = 0.612 for teachers, and *α* = 0.797 for principals. It’s worth noting that since students only answered one item, there was no need to assess reliability for their responses.

The perceived stress scale (PSS-4) (Warttig et al., 2013), a highly validated instrument for measuring stress, was incorporated and evaluated using four items among students, parents, teachers, and principals. These items gauged how frequently certain feelings and thoughts were experienced by each stakeholder in the past month. It’s important to note that the questions were identical, although tailored to each specific group. To assess the scale’s reliability, Cronbach’s Alpha was employed, yielding the following results for each of the four stakeholder groups: *α* = 0.668 for students, *α* = 0.726 for parents, *α* = 0.655 for teachers, and *α* = 0.683 for principals.

Psychosocial aspects during remote learning were evaluated using three items for students and parents, and five items for teachers and principals. These items encompassed the attitudes of all stakeholders toward remote learning. Three of the questions were identical, albeit customized for each specific group. To gauge the scale’s reliability, Cronbach’s Alpha was applied, resulting in the following coefficients for each of the four stakeholder groups: *α* = 0.819 for students, *α* = 0.793 for parents, *α* = 0.746 for teachers, and *α* = 0.615 for principals.

The evaluation of psychosocial aspects during frontal learning involved the use of 2 items for students and parents, and 5 items for teachers and principals. These items captured various facets of the attitudes of all stakeholders toward in-person learning. Two of the questions were the same, though tailored to each specific group. The reliability of the scale was assessed using Cronbach’s Alpha, resulting in the following coefficients for each of the four stakeholder groups: *α* = 0.503 for students, *α* = 0.524 for parents, *α* = 0.746 for teachers, and *α* = 0.883 for principals.

Digital literacy was assessed by 2 items among students and parents, and 6 items among teachers and principals. The components of this index encompass competencies and preparedness with remote instruction/ learning among all the stakeholders. Two of the questions were identical (though adapted to each specific population). The scale reliability was measured by Alpha Cronbach and results for each of the four stakeholders were (*α* = 0.666) among students, (*α* = 0.754) among parents, (*α* = 0.777) among teachers, and (*α* = 0.759) among principals.

Pedagogic support was assessed by 2 items among all four stakeholders. The components of this index encompass support to the stakeholders (including technical and academic guidance) during both distance and frontal learning/ teaching. All the questions were identical (though adapted to each specific population). The scale reliability was measured by Alpha Cronbach and results for each of the four stakeholders were (*α* = 0.662) among students, (*α* = 0.720) among parents, (*α* = 0.80) among teachers, and (*α* = 0.826) among principals.

Resources were assessed by 2 items all four stakeholders. The components of this index encompass availability of resources (including teaching and learning materials, time, etc.) during both distance and frontal learning/ teaching. All the questions were identical (though adapted to each specific population). The scale reliability was measured by Alpha Cronbach and results for each of the four stakeholders were (*α* = 0.543) among students, (*α* = 0.504) among parents, (*α* = 0.698) among teachers, and (*α* = 0.538) among principals.

Infrastructure was assessed by 3 items among all four stakeholders. The components of this index encompass availability of infrastructure needed for learning/ teaching (including the availability of internet, software, digital infrastructure, physical space, etc.) during both distance and frontal learning/ teaching. All the questions were identical (though adapted to each specific population). The scale reliability was measured by Alpha Cronbach and results for each of the four stakeholders were (*α* = 0.802) among students, (*α* = 0.795) among parents, (*α* = 0.813) among teachers, and (*α* = 0.665) among principals.

Distant versus frontal teaching/ learning was assessed by 4 items among all the stakeholders. The components of this index encompass attitudes toward differences between distance and frontal learning with respect to resources, pedagogical support, quality of communication, and psychosocial aspects. All the questions were identical (though adapted to each specific population). The scale reliability was measured by Alpha Cronbach and results for each of the four stakeholders were (*α* = 0.853) among students, (*α* = 0.833) among parents, (*α* = 0.894) among teachers, and (*α* = 0.858) among principals.

Perceived functional resilience was assessed by 13 items among students and parents, and 19 items among teachers and principals. The components of this index encompass attitudes toward functioning through the distance learning phase during the COVID-19 crisis, the ability of schools to take away lessons learned and adapt them from the COVID-19 school closures, and preparedness for future adversities. Thirteen of the questions were identical (though adapted to each specific population). The scale reliability was measured by Alpha Cronbach and results for each of the four stakeholders were (*α* = 0.925) among students, (*α* = 0.726) among parents, (*α* = 0.949) among teachers, and (*α* = 0.944) among principals. Initially, the composite index did not include the perceived functional resilience index score, however following assessment of Alpha Cronbach both before inclusion (*α* = 0.726) and after inclusion (*α* = 0.925), the perceived functional resilience index was integrated into the composite score.

#### 3.1.1 Demographics

Demographic information for students was gathered through 11 items, encompassing details such as gender, birth year, place of residence, the number of children under 18 in the same household, the number of dependents over 18 in the same household, religion, level of religiosity, school type, school location, grade, and class size. In addition to these factors, demographic data collected for parents, teachers, and principals also encompassed marital status, educational attainment, and income level.

An example of the translated tool in English for students is provided in Appendix 1.

### 3.2 Results of modified Delphi process regarding the relative importance of each component/ stakeholder to the composite functional resilience index

The modified eDelphi process was conducted with 20 invited content experts, among them 17 responded during the first round of the Delphi (85% response rate), and 15 had responded (88.2% response rate) during the second round. The professional background experience of the respondents includes participants from educational sciences (3 of 17 respondents, 17.6%); academia, resilience and psychosocial systems (7 of 17 respondents, 41.1%); government, Ministry of Education (2 of 17 respondents, 11.8%); and school professionals (6 of 17 respondents, 35.2%), including 5 teachers and one principal. 64.7% of the modified eDelphi participants were female. The aim was to achieve consensus among experts concerning the relative importance of each of the components to the functional resilience index. Table 1 presents the average score of all respondents for round one and two, as well as the level of agreement achieved. Fleiss kappa during round 1 for overall agreement was 0.076, with standard error of 0.020. During round 2, Fleiss kappa increased to 0.566 with standard error of 0.026.

**Table 1:** Ranking of the relative importance of the functional resilience categories (the average score) and the extent of agreement of the content experts with respect to each of the categories.

Category	School communication	Stress Perception Scale (PSS)	Psychosocial Aspects	Digital Literacy	Pedagogical Support	Resources	Infrastructure	Perceived Functional Resilience	Distance versus Frontal learning/ teaching
The average score of each category -Round 1 (*n* = 17)	15%	10%	12%	7%	14%	14%	11%	12%	5%
Level of agreement – Round 1	41.2%	47.1%	41.2%	52.9%	29.4%	52.9%	52.9%	35.3%	64.7%
The average score of each category -Round 2 (*n* = 15)	15%	10%	12%	7%	14%	14%	12%	12%	4%
Level of agreement- Round 2	80%	73.3%	73.3%	93.3%	73.3%	80%	80%	73.3%	80%

In addition, the aim was to achieve consensus among experts concerning the relative importance of each stakeholder to the functional resilience index. Table 2 presents the average score of all respondents for rounds one and two, as well as the levels of agreement achieved. Fleiss kappa during round 1 for overall agreement was 0.037, with standard error of 0.023. During round 2, Fleiss kappa increased to 0.702 with standard error of 0.034.

**Table 2:** Ranking of the relative importance of the four key stakeholders to the functional resilience of the school (the average score) and the extent of agreement of the content experts with respect to each stakeholder category.

Category	Students	Parents	Teachers	Principals
The average score of each category -round 1 (*n* = 17)	29%	16%	31%	24%
Level of agreement – round 1	47.1%	41.2%	52.9%	35.3%
The average score of each category -round 2 (*n* = 15)	28%	15%	32%	25%
Level of agreement- round 2	73.3%	80%	86.6%	73.3%

### 3.3 Utilizing the tool to assess functional resilience of schools

The newly developed tool was utilized to gather data from the four stakeholder groups across various schools.

#### 3.3.1 Participants and characteristics of the school sample

A total of 1802 participants were included in the study, comprising 1,000 students, 301 parents, 449 teachers, and 52 principals. The same sample dataset here is explored as in Kaim et al. (2023).^2^ Table 3 displays the demographic characteristics of all four stakeholder groups in the surveyed population. The students’ average age was 16.7 years, with a majority (52.5%) being male. Parents had an average age of 48.0 years, with the majority being female (68.4%). Teachers had an average age of 41.6 years, with the majority being female (80.8%). Finally, the average age of principals was 46.7 years, with the majority being female (55.8%).

**Table 3:** Demographic traits categorized by the four stakeholder groups, namely Students, Parents, Teachers, and Principals (see Kaim et al., 2023).

	Total *n* = 1802	Students *n* = 1,000	Parents *n* = 301	Teachers *n* = 449	Principals *n* = 52	*p*-Value
Gender						
Male	40.4	52.5	31.6	19.2	44.2	<0.001
Female	59.6	47.5	68.4	80.8	55.8	
Age (Mean ± SD)	29.4 ± 15.4	16. 7 ± 0.8	48.0 ± 5.1	41.6 ± 11.8	46.7 ± 8.9	<0.001*

Same population is utilized as in the Kaim et al. (2023) study.

Following the modified eDelphi results, the components were weighted according to the delineated importance of each variable as well as the relative importance of each stakeholder, to recompute a weighed composite functional resilience index score.

A sample of 890 Jewish schools in Israel was included in the study, with 67.1% (*n* = 597) being state schools and 32.9% (*n* = 293) being religious schools. The largest proportion of sampled schools, accounting for 29.6% (*n* = 261), were located in the Central region of Israel. The schools included in the sample are similarly those featured in the Kaim et al. (2023) research.

#### 3.3.2 Indexes and characteristics of differences

Table 4 exhibits the mean values for each index, showcasing distinctions among the four stakeholder groups. Based on the Bonferroni multiple comparisons test, the analysis unveiled the following noteworthy disparities: Concerning the Distance Learning (DL) communication index, statistically significant differences were discerned between students and parents (*p* < 0.001), students and teachers (*p* < 0.001), parents and principals (*p* = 0.001), and parents and teachers (*p* < 0.001); Regarding the Communication Frontal Learning (FL) index, notable differences emerged between students and teachers (*p* < 0.001), parents and principals (*p* < 0.01), and parents and teachers (*p* < 0.001). In terms of the PSS index, significant distinctions were evident between students and parents (*p* < 0.001), as well as between students and teachers (*p* < 0.001).

**Table 4:** Differences between the four stakeholders with respect to the indexes.

Index	Mean ± SD	Students *n* = 1,000	Parents *n* = 301	Teachers *n* = 449	Principals *n* = 52	*p*-Value
Communication distance learning (DL)^1^		3.30 ± 0.93^1^	4.06 ± 0.60^1^	3.61 ± 0.77^1^	3.57 ± 0.66^1^	<0.001
Communication frontal learning (FL)^1^		3.83 ± 0.94^1^	3.70 ± 0.81^1^	4.18 ± 0.68^1^	4.15 ± 0.75^1^	<0.001
Perceived stress scale (PSS)^1^		3.35 ± 0.72^1^	3.65 ± 0.68^1^	3.57 ± 0.70^1^	3.50 ± 0.73^1^	<0.001
Psychosocial aspects distance learning (DL)^1^		2.87 ± 1.10^1^	2.57 ± 1.04^1^	3.26 ± 0.83^1^	3.06 ± 0.64^1^	<0.001
Psychosocial aspects frontal learning (FL)^1^		3.41 ± 0.85^1^	3.64 ± 0.84^1^	4.03 ± 0.64^1^	4.45 ± 0.1.14^1^	<0.001
Digital literacy		3.34 ± 0.92	3.47 ± 1.02	3.61 ± 0.73	3.62 ± 0.67	<0.001
Pedagogic support		3.18 ± 0.96	3.12 ± 1.01	2.91 ± 1.03	3.33 ± 0.87	<0.001
Infrastructure		4.26 ± 0.80	4.52 ± 0.69	3.63 ± 0.95	4.05 ± 0.90	<0.001
Perceived functional resilience		3.39 ± 0.76	3.49 ± 0.86	3.83 ± 0.72	3.76 ± 0.70	<0.001
Distance VS. frontal learning		2.71 ± 0.95	2.46 ± 0.87	2.78 ± 0.94	2.46 ± 0.85	<0.001
Stakeholder support- distance*					3.33 ± 0.64	
Stakeholder support- frontal*					3.33 ± 0.70	
Composite functional resilience index	3.44 ± 0.51	3.35 ± 0.50	3.48 ± 0.47	3.59 ± 0.52	3.55 ± 0.47	<0.001
Weighted composite functional resilience index	3.53 ± 0.52	3.39 ± 0.51	3.56 ± 0.49	3.59 ± 0.47	3.61 ± 0.55	<0.001

Data presented by Mean ± SD.

*Only applicable to principals.

^1^Data previously displayed in Kaim et al. (2023) pertaining to Communication DL and FL, Psychosocial Aspects DL and FL, as well as PSS for the four respective stakeholders.

No significant differences according to the various school characteristics (type, school region, size) were found (see Table 5).

**Table 5:** Differences according to characteristics of school for the composite functional resilience score.

	Composite Functional Resilience Score	*f*-Value	*p*-Value
Type of school (*n* = 890)			
State school (*n* = 597)	3.42 ± 0.51	−1.663	0.097
Religious school (*n* = 293)	3.47 ± 0.50		
School region			
North (*n* = 115)	3.53 ± 0.53	1.309	0.250
Haifa (*n* = 93)	3.43 ± 0.46		
Tel Aviv (*n* = 120)	3.41 ± 0.55		
Center (*n* = 261)	3.43 ± 0.50		
Jerusalem (*n* = 87)	3.41 ± 0.50		
South (*n* = 140)	3.43 ± 0.53		
West Bank(*n* = 65)	3.46 ± 0.41		
Gaza Envelope Region (within 40 km)			
Yes (*n* = 115)	3.42 ± 0.53	0.500	0.617
No (*n* = 765)	3.44 ± 0.51		
Number of students in school			
100 and below (*n* = 161)	3.49 ± 0.52	0.412	0.744
101–200 (*n* = 187)	3.48 ± 0.52		
201–500 (*n* = 251)	3.46 ± 0.48		
500 and above (*n* = 151)	3.45 ± 0.53		

Data presented by Mean ± SD.

Concerning the Psychosocial Aspects Distance Learning (DL) index, significant variations were observed between students and parents (*p* < 0.001), students and teachers (*p* < 0.001), parents and teachers (*p* < 0.001), and principals and parents (*p* < 0.01); In the context of the Psychosocial Aspects Frontal Learning (FL) index, there were significant distinctions noted between students and parents (*p* < 0.001), students and teachers (*p* < 0.001), students and principals (*p* < 0.001), parents and teachers (*p* < 0.001), parents and principals (*p* < 0.01), and principals and teachers (*p* < 0.01); For the Digital Literacy index, noteworthy differences were observed solely between students and teachers (*p* < 0.001); Regarding Pedagogic Support, significant variations were evident between students and teachers (*p* < 0.001), parents and teachers (*p* < 0.05), and teachers and principals (*p* < 0.05). Regarding the Infrastructure Index, there were significant distinctions observed between students and parents (*p* < 0.001), students and principals (*p* < 0.001), students and teachers (*p* < 0.001), parents and principals (*p* < 0.001), parents and teachers (*p* < 0.001), and teachers and principals (*p* < 0.01); In terms of Perceived Functional Resilience, noteworthy differences emerged between students and teachers (*p* < 0.001), students and principals (*p* < 0.001), parents and principals (*p* < 0.05), and parents and teachers (*p* < 0.001); When considering Distant vs. Frontal Learning, significant variations were evident between students and parents (*p* < 0.001), as well as between students and teachers (*p* < 0.001); For the overall Composite Functional Resilience Index, significant differences were noted between students and parents (*p* < 0.001), students and teachers (*p* < 0.001), and parents and teachers (*p* < 0.05); Lastly, for the Weighted Composite Functional Resilience Score, significant distinctions were found between students and parents (*p* < 0.001), and students and teachers (*p* < 0.001).

## 4 Discussion

In our earlier research using the same dataset (Kaim et al., 2023), we explored the perceived levels of communication and psychosocial aspects among high school students, parents, teachers, and principals during both distance and frontal learning in the Israeli education system. The results highlighted significant consequences of distance learning on communication and psychosocial aspects, resulting in enduring long-term distress, especially among students. In our present study, we aimed to extend beyond individual indicators and instead identify key indicators for assessing the overall functional resilience of school systems. We developed a structured tool to periodically evaluate fluctuations, with the ultimate goal of enhancing the response and resilience of school systems to all challenges. To ensure improved response and resilience of school systems to the challenges posed by the COVID-19 pandemic and beyond, it is imperative to establish comprehensive, structured assessments. The performance success of any system, i.e., the continuity of the system’s functional services both during times of normalcy, as well as during and after crises, depends on a variety of factors. In the context of the education system, the COVID-19 pandemic has exposed its vulnerabilities worldwide (Krishnamoorthy and Keating, 2021; Leo et al., 2021; Manivannan et al., 2021). The disruption and subsequent reintegration of the global school system highlight the importance of ensuring that schools are functionally resilient during times of crisis, both in traditional and remote learning environments.

This study makes several contributions to the field. Firstly, it provides a conceptual framework of what constitutes key characteristics of the functionality of a school system. This was conducted via an extensive literature review examining the key challenges faced by the education system throughout the covid-19 school closure periods, among varied key stakeholders. To date, to the authors’ knowledge, there has been only limited examination of the conceptual framework of functional resilience and no studies specifically examined the school system. The resilience of individual stakeholders such as students, parents, teachers, and administrators has been studied (Cusinato et al., 2020; Zadok-Gurman et al., 2021; Sorkkila and Aunola, 2022), however, there are gaps in examining the integration of crucial stakeholders’ attitudes, roles, and performance when assessing the systemic resilience. The most critical components of the functional resilience of a school system among all four stakeholders that were determined in this study (communication, psychosocial factors, digital proficiency, pedagogical support, infrastructure, attitudes toward remote and in-person learning, stakeholder support, and perceived functional resilience), are in line with previous work, despite not previously being identified through a collective conceptualization of functional resilience (Haycock, 2007; Sun et al., 2008; Sheppard and Dibbon, 2011; Arifin, 2015; Kuzminskyi et al., 2019). Based on the conceptual framework, the developed tailor-made tool provides educational authorities (local, regional, or national) as well as individual schools, the ability to systematically examine their resilience, the attitudes of the various stakeholders, and the overall perception of their levels of preparedness for future crises. The application of the developed tool among 890 Israeli schools was instrumental in indicating differences among stakeholders, as well as parameters / components where further interventions may be needed as part of the ongoing effort to maintain and improve the functional resilience of schools, both during routine times and adversities. The assessment tool that was developed contributes toward the strengthening of the education system as it provides benchmarks for maintaining efficient functioning. Furthermore, it facilitates the activation of robust monitoring systems that will enable the identification of strengths and weaknesses within the system, to accordingly improve gaps. While our results are exploratory and further long-term research is needed, this tool can be adopted and tailored to various school systems throughout various regions beyond Israel, as well as to various organizational systems, to enhance their levels of functional resilience and ensure their preparedness for potential adversities.

## 5 Limitations

A primary limitation of this study is the absence of established tools specifically designed for the assessment of functional resilience, particularly within school systems. This made it challenging to benchmark the proposed tool against any recognized golden standard. Additionally, our reliance on an internet panel for data collection means our results might only reflect views of those with digital literacy. This method may also constrain the depth of insights regarding relationships between stakeholders. While structured, quantitative questionnaires like ours can introduce a social desirability bias, we attempted to counteract this by employing a large sample size. This study also did not explore the unique issues encountered by schools serving specialized populations, such as children with disabilities—a facet worthy of future exploration. Moreover, the research focused on Jewish Israeli participants, excluding other vital groups like the Arab minority in Israel. Future studies should consider and encompass these diverse populations for a more comprehensive understanding.

## 6 Conclusion

The study developed a comprehensive tool for the assessment of the functional resilience of school systems. To enhance the resilience and preparedness for future adversities that school systems may encounter beyond the COVID-19 pandemic, such as natural (e.g., earthquakes) and manmade disasters (e.g., conflicts), an assessment based on a structured tool should be incorporated and conducted periodically. The process of evaluation will not only facilitate lessons learned regarding the ability of the school system to adapt to future adversities but can also assist in identifying vulnerabilities (for example, among specific stakeholders) and thus enable tailored interventions to be designed to the specific needs of each school. It is highly recommended that such a tool be implemented across schools throughout various regions of the world, given that the current study was limited to schools in Israel. This would help determine the tool’s generalizability and whether the tool can be applied on a global scale.

## Data availability statement

The raw data supporting the conclusions of this article will be made available by the authors, without undue reservation.

## Ethics statement

The studies involving humans were approved by the Tel Aviv University/ Ministry of Education Israel. The studies were conducted in accordance with the local legislation and institutional requirements. Written informed consent for participation in this study was provided by the participants’ legal guardians/next of kin.

## Author contributions

AK: Conceptualization, Data curation, Formal analysis, Funding acquisition, Investigation, Methodology, Project administration, Validation, Writing – original draft. MS-T: Data curation, Formal analysis, Writing – review & editing. BA: Conceptualization, Funding acquisition, Methodology, Project administration, Writing – review & editing. SL-a: Conceptualization, Funding acquisition, Writing – review & editing, Project administration.

## Footnotes

^1^http://www.ipanel.co.il

^2^see https://www.mdpi.com/1660-4601/20/6/4837.

## References

[ref2] Arifin H. M. (2015). The influence of competence, motivation, and organisational culture to high school teacher job satisfaction and performance. Int. Educ. Stud. 8, 38–45. doi: 10.5539/ies.v8n1p38

[ref3] Armitage R. Nellums L. B. (2020). Considering inequalities in the school closure response to COVID-19. The lancet. Glob. Health 8:e644. doi: 10.1016/S2214-109X(20)30116-9, PMID: 32222161 PMC7195275

[ref4] Azevedo J. P. Hasan A. Goldemberg D. Geven K. Iqbal S. A. (2021). Simulating the potential impacts of COVID-19 school closures on schooling and learning outcomes: a set of global estimates. World Bank Res. Obs. 36, 1–40.

[ref5] Biggs R. Schlüter M. Biggs D. Bohensky E. L. BurnSilver S. Cundill G.. (2012). Toward principles for enhancing the resilience of ecosystem services. Annu. Rev. Environ. Resour. 37, 421–448. doi: 10.1146/annurev-environ-051211-123836

[ref6] Bryce C. Ring P. Ashby S. Wardman J. K. (2020). Resilience in the face of uncertainty: early lessons from the COVID-19 pandemic. J. Risk Res. 23, 880–887. doi: 10.1080/13669877.2020.1756379

[ref7] Colao A. Piscitelli P. Pulimeno M. Colazzo S. Miani A. Giannini S. (2020). Rethinking the role of the school after COVID-19. Lancet Public Health 5:e370. doi: 10.1016/S2468-2667(20)30124-9 32464100 PMC7247785

[ref9] Contini D. Di Tommaso M. L. Muratori C. Piazzalunga D. Schiavon L. (2021). *The COVID-19 pandemic and school closure: learning loss in mathematics in primary education* (no. 664). Collegio Carlo Alberto. 1–32. doi: 10.2139/ssrn.4114323

[ref10] Cusinato M. Iannattone S. Spoto A. Poli M. Moretti C. Gatta M.. (2020). Stress, resilience, and well-being in Italian children and their parents during the COVID-19 pandemic. Int. J. Environ. Res. Public Health 17:8297. doi: 10.3390/ijerph17228297, PMID: 33182661 PMC7696524

[ref11] Daniel J. (2020). Education and the COVID-19 pandemic. Prospects 49, 91–96. doi: 10.1007/s11125-020-09464-3, PMID: 32313309 PMC7167396

[ref12] Dooley D. G. Bandealy A. Tschudy M. M. (2020). Low-income children and coronavirus disease 2019 (COVID-19) in the US. JAMA Pediatr. 174, 922–923. doi: 10.1001/jamapediatrics.2020.2065 32401283

[ref13] Doyle O. (2020). COVID-19: exacerbating educational inequalities. *Public Policy*. Available at: https://efaidnbmnnnibpcajpcglclefindmkaj/https://public policy.ie/downloads/papers/2020/COVID_19_Exacerbating_Educational_Inequalities.pdf

[ref14] Donitsa-Schmidt S. Ramot R. (2020). Opportunities and challenges: teacher education in Israel in the Covid-19 pandemic. J. Educ. Teach. 46, 586–595. doi: 10.1080/02607476.2020.1799708

[ref15] Flack C. B. Walker L. Bickerstaff A. Earle H. Margetts C. (2020). Educator perspectives on the impact of COVID-19 on teaching and learning in Australia and New Zealand. Melbourne, Australia: Pivot Professional Learning.

[ref16] Flack CB Walker L Bickerstaff A Earle H Johnson CL. (2021). Principal perspectives on the impact of COVID-19: Pathways toward equity in Australian schools. Melbourne, Australia: Pivot Professional Learning.

[ref17] Gröschke D. Hofmann E. Müller N. D. Wolf J. (2022). Individual and organizational resilience—insights from healthcare providers in Germany during the COVID-19 pandemic. Front. Psychol. 13:965380. doi: 10.3389/fpsyg.2022.965380 36092080 PMC9453859

[ref18] Grooms A. A. Childs J. (2021). “We need to do better by kids”: changing routines in US schools in response to COVID-19 school closures. J. Educ. Stud. Placed Risk 26, 135–156. doi: 10.1080/10824669.2021.1906251

[ref20] Haycock K. (2007). Collaboration: critical success factors for student learning. Sch. Libr. Worldw. 13:25.

[ref21] Kaden U. (2020). COVID-19 school closure-related changes to the professional life of a K–12 teacher. Educ. Sci. 10:165. doi: 10.3390/educsci10060165

[ref22] Kaim A. Lev-Ari S. Adini B. (2023). Distress following the COVID-19 pandemic among schools’ stakeholders: psychosocial aspects and communication. Int. J. Environ. Res. Public Health 20:4837. doi: 10.3390/ijerph20064837 36981747 PMC10049332

[ref23] Krishnamoorthy R. Keating K. (2021). Education crisis, workforce preparedness, and COVID-19: reflections and recommendations. Am. J. Econ. Sociol. 80, 253–274. doi: 10.1111/ajes.12376, PMID: 34230670 PMC8250606

[ref24] Kuzminskyi A. I. Bida O. A. Kuchap O. V. Yezhova O. V. Kuchai T. P. (2019). Information support of educationalists as an important function of a postgraduate education system. Roman. J. Multidimen. Educ. 11, 263–279. doi: 10.18662/rrem/150

[ref25] Leo S. Alsharari N. M. Abbas J. Alshurideh M. T. (2021). “From offline to online learning: a qualitative study of challenges and opportunities as a response to the COVID-19 pandemic in the UAE higher education context” in The effect of coronavirus disease (COVID-19) on business intelligence. ed. Kacprzyk J. (Cham: Springer), 203–217.

[ref26] Loosemore M. Chow V. Harvison T. (2013). Inter-agency governance risk in managing hospital responses to extreme weather events in New South Wales, Australia: a facilities management perspective of shared situational awareness. Constr. Manag. Econ. 31, 1072–1082. doi: 10.1080/01446193.2013.853128

[ref27] Lugo D. (2022). The Effects of Virtual Learning during the COVID-19 Pandemic on Teachers and Students in Grades K-12 within the United States: a systematic review (Doctoral dissertation, Cedar Crest College). 1–57.

[ref28] Mahmoudi F. Mohamed S. (2018a). “A review of hospitals functional resilience and performance indicators” in The tenth international conference on construction in the 21st century (CITC-10) (Colombo, Sri Lanka).

[ref29] Mahmoudi F. Mohamed S. (2018b). Modelling hospitals functional performance using MICMAC analysis: a resilience perspective. In *1st International Conference on Construction Project Management and Construction Engineering*.

[ref30] Manivannan M. Jogalekar M. P. Kavitha M. S. Maran B. A. Gangadaran P. (2021). A mini-review on the effects of COVID-19 on younger individuals. Exp. Biol. Med. 246, 293–297. doi: 10.1177/1535370220975118, PMID: 33210552 PMC7859671

[ref31] Masten A. S. (2018). Resilience theory and research on children and families: past, present, and promise. J. Fam. Theory Rev. 10, 12–31. doi: 10.1111/jftr.12255

[ref32] Paltiel O. Hochner H. Chinitz D. Clarfield A. M. Gileles-Hillel A. Lahad A.. (2021). Academic activism on behalf of children during the COVID-19 pandemic in Israel; beyond public health advocacy. Israel J. Health Policy Res. 10, 1–13. doi: 10.1186/s13584-021-00485-7 PMC8371603 34407864

[ref33] Pollock K. (2020). School leaders’ work during the COVID-19 pandemic: a two-pronged approach. Int. Stud. Educ. Adm. 48:38.

[ref34] Prime H. Wade M. Browne D. T. (2020). Risk and resilience in family well-being during the COVID-19 pandemic. Am. Psychol. 75, 631–643. doi: 10.1037/amp0000660 32437181

[ref9001] Psacharopoulos G. Collis V. Patrinos H. A. Vegas E. (2021). The COVID-19 Cost of School Closures in Earnings and Income across the World. Comp. Educ. Rev. 65, 271–287.

[ref35] Rautela P. Joshi G. C. Bhaisora B. (2011). Seismic vulnerability of the health infrastructure in the Himalayan township of Mussoorie, Uttarakhand, India. Int. J. Disaster Resil. Built Environ. 2, 200–209. doi: 10.1108/17595901111167088

[ref36] Sheppard B. Dibbon D. (2011). Improving the capacity of school system leaders and teachers to design productive learning environments. Leadersh. Policy Sch. 10, 125–144. doi: 10.1080/15700763.2010.502610

[ref37] Sorkkila M. Aunola K. (2022). Resilience and parental burnout among Finnish parents during the COVID-19 pandemic: variable and person-oriented approaches. Fam. J. 30, 139–147. doi: 10.1177/10664807211027307, PMID: 35399756 PMC8980848

[ref38] Stein-Zamir C. Abramson N. Shoob H. Libal E. Bitan M. Cardash T.. (2020). A large COVID-19 outbreak in a high school 10 days after schools’ reopening, Israel, May 2020. Eur. Secur. 25:2001352. doi: 10.2807/1560-7917.ES.2020.25.29.2001352 PMC7384285 32720636

[ref39] Sun P. C. Tsai R. J. Finger G. Chen Y. Y. Yeh D. (2008). What drives a successful e-learning? An empirical investigation of the critical factors influencing learner satisfaction. Comput. Educ. 50, 1183–1202. doi: 10.1016/j.compedu.2006.11.007

[ref40] Tso W. W. Wong R. S. Tung K. T. Rao N. Fu K. W. Yam J. C.. (2020). Vulnerability and resilience in children during the COVID-19 pandemic. Eur. Child Adolesc. Psychiatry 31, 161–176. doi: 10.1007/s00787-020-01680-8 33205284 PMC7671186

[ref1] UNESCO. (2020). UN secretary-general warns of education catastrophe, pointing to UNESCO estimate of 24 million learners at risk of dropping out. Available at: https://en.unesco.org/news/secretary-general-warns-education-catastrophe-pointing-unesco-estimate-24-million-learners-0 (Accessed December 4, 2021).

[ref41] Warttig S. L. Forshaw M. J. South J. White A. K. (2013). New, normative, English-sample data for the short form perceived stress scale (PSS-4). J. Health Psychol. 18, 1617–1628. doi: 10.1177/1359105313508346, PMID: 24155195

[ref42] Zadok-Gurman T. Jakobovich R. Dvash E. Zafrani K. Rolnik B. Ganz A. B.. (2021). Effect of inquiry-based stress reduction (IBSR) intervention on well-being, resilience and burnout of teachers during the COVID-19 pandemic. Int. J. Environ. Res. Public Health 18:3689. doi: 10.3390/ijerph18073689, PMID: 33916258 PMC8037267

[ref43] Zhao Y. (2020). COVID-19 as a catalyst for educational change. Prospects 49, 29–33. doi: 10.1007/s11125-020-09477-y, PMID: 32836421 PMC7287028

[ref44] Zhong S. Clark M. Hou X. Y. Zang Y. FitzGerald G. (2015). Development of key indicators of hospital resilience: a modified Delphi study. J. Health Serv. Res. Policy 20, 74–82. doi: 10.1177/1355819614561537, PMID: 25504827

