# Supplementary material for: An innovative tool to assess the functional resilience of a school system: learning from the COVID-19 pandemic
Source: Front Psychol. 2023 Nov 24;14:1291621. doi: 10.3389/fpsyg.2023.1291621 (PMC10706003; doi:10.3389/fpsyg.2023.1291621)
Supplement: Supplementary file 1 [file Data_Sheet_1.docx]

Appendix 1.

Functional resilience translated measurement tool – Student population.

**To what extent do you agree or disagree with the following:**

|  | **Agree to a very large extent** | **Largely agree** | **Moderately agree** | **Strongly disagree** | **Disagree to a very large extent** |
| --- | --- | --- | --- | --- | --- |
| 1.I have internet available and accessible should I need to learn remotely | 5 | 4 | 3 | 2 | 1 |
| 2. I have the necessary means for distance learning such as computer and software (such as Zoom, Google Class) | 5 | 4 | 3 | 2 | 1 |
| 3. I have the option to learn remotely in a quiet and adapted place | 5 | 4 | 3 | 2 | 1 |
| 4. When I learn remotely, I have difficulty separating learning tasks from other tasks, such as helping family members | 5 | 4 | 3 | 2 | 1 |
| 5. When learning in the classroom, I have difficulty separating learning tasks from other tasks, such as helping family members | 5 | 4 | 3 | 2 | 1 |
| 6. I have readily available and effective learning materials (such as documents, films, and courseware) for both classroom and distance learning | 5 | 4 | 3 | 2 | 1 |
| 7. The school provides me with technical assistance to the extent required during classroom and distance learning | 5 | 4 | 3 | 2 | 1 |
| 8. My teachers' availability and accessibility about me is maintained to a similar extent during distance learning compared to classroom learning | 5 | 4 | 3 | 2 | 1 |
| 9. The school as a system is currently prepared and able to carry out learning in the classroom in a good way | 5 | 4 | 3 | 2 | 1 |
| 10. The school as a system is now ready and able to perform distance learning well | 5 | 4 | 3 | 2 | 1 |

**To what extent do you agree or disagree with the following statements relating to the period when the school was closed and learning was throughdistance learning:**

| **During the period when the school was closed and learning was throughdistance learning:** | **Agree to a very large extent** | **Largely agree** | **Moderately agree** | **Strongly disagree** | **Disagree to a very large extent** |
| --- | --- | --- | --- | --- | --- |
| 11. I had the opportunity to make personal contact with the teachers and school staff | 5 | 4 | 3 | 2 | 1 |
| 12. My teachers kept in regular contact with me | 5 | 4 | 3 | 2 | 1 |
| 13. The teachers monitored my progress to the required extent | 5 | 4 | 3 | 2 | 1 |
| 14. I enjoy remote learning | 5 | 4 | 3 | 2 | 1 |
| 15. I am highly motivated to learn remotely | 5 | 4 | 3 | 2 | 1 |
| 16. Distance learning wears me out | 5 | 4 | 3 | 2 | 1 |

**To what extent do you agree or disagree with the following sentences relating to classroom learning:**

|  | **Agree to a very large extent** | **Largely agree** | **Moderately agree** | **Strongly disagree** | **Disagree to a very large extent** |
| --- | --- | --- | --- | --- | --- |
| 17. In classroom learning, I keep in regular contact with my teachers | 5 | 4 | 3 | 2 | 1 |
| 18. In classroom learning, I am highly motivated to learn | 5 | 4 | 3 | 2 | 1 |
| 19. Classroom learning is a heavy burden | 5 | 4 | 3 | 2 | 1 |

**The following questionsrelate to your feelings and thoughts over the past month. Please select the most appropriate option.**

| **Very often** | **Quiteoften** | **sometimes** | **Rarely** | **never** |  |
| --- | --- | --- | --- | --- | --- |
| 5 | 4 | 3 | 2 | 1 | 20. In the past month, how often have you felt out of control over the important things in your life? |
| 5 | 4 | 3 | 2 | 1 | 21. In the past month, how often have you felt confident in your ability to handle your personal problems? |
| 5 | 4 | 3 | 2 | 1 | 22. In the past month, how often have you felt that things are evolving according to your wishes? |
| 5 | 4 | 3 | 2 | 1 | 23. In the past month, how often have you felt the difficulties accumulating to the point that you couldn't overcome them? |

**To what extent do you agree or disagree with the following sentences relating to the school you attend?**

|  | **Agree to a very large extent** | **Largely agree** | **Agree if**  **Medium** | **Strongly disagree** | **Disagree to a very large extent** |
| --- | --- | --- | --- | --- | --- |
| 24. My school functioned properly during the COVID-19 crisis while there was distance learning | 5 | 4 | 3 | 2 | 1 |
| 25. In my school there is mutual help and concern between the students | 5 | 4 | 3 | 2 | 1 |
| 26. My school is organized for emergencies, including when students do not come to school. | 5 | 4 | 3 | 2 | 1 |
| 27. I am proud to tell others what school I attend. | 5 | 4 | 3 | 2 | 1 |
| 28. The relationship between the staff members at my school is good | 5 | 4 | 3 | 2 | 1 |
| 29. I have great confidence in the principal and management team of my school | 5 | 4 | 3 | 2 | 1 |
| 30. I can count on the staff at my school to come to my aid in case of a crisis | 5 | 4 | 3 | 2 | 1 |
| 31.My school staff knows their role in an emergency. | 5 | 4 | 3 | 2 | 1 |
| 32. I feel like I belong to my school community | 5 | 4 | 3 | 2 | 1 |
| 33.My school conducts discussions and exercises relating to the school's functioning in times of emergency. | 5 | 4 | 3 | 2 | 1 |
| 34. My school has successfully passed the back-to-school phase after distance learning | 5 | 4 | 3 | 2 | 1 |
| 35. My school takes the necessary steps to help us (the students) cope with the difficulties created by isolation and distancing | 5 | 4 | 3 | 2 | 1 |
| 36. Teachers have the ability to give us (students) mental first aid | 5 | 4 | 3 | 2 | 1 |

**To what extent do you agree with the following statementsin the context ofdistance learning compared to classroom learning?**

|  | **Agree to a very large extent** | **Largely agree** | **Agree if**  **Medium** | **Strongly disagree** | **Disagree to a very large extent** |
| --- | --- | --- | --- | --- | --- |
| 37. The resources available to me for distance learning were better compared to classroom learning | 5 | 4 | 3 | 2 | 1 |
| 38. The level of pedagogical support I received for distance learning was better compared to classroom learning | 5 | 4 | 3 | 2 | 1 |
| 39. The quality of communication between myself and the teachers and school staff in distance learning was better compared to classroom learning | 5 | 4 | 3 | 2 | 1 |
| 40. My level of motivation to learn remotely was higher compared to classroom learning | 5 | 4 | 3 | 2 | 1 |
